# Ion irradiation induced structural modifications and increase in elastic modulus of silica based thin films

**DOI:** 10.1038/srep40100

**Published:** 2017-01-10

**Authors:** S. A. Shojaee, Y. Qi, Y. Q. Wang, A. Mehner, D. A. Lucca

**Affiliations:** 1 School of Mechanical and Aerospace Engineering, 218 Engineering North, Oklahoma State University, Stillwater, OK 74078, USA; 2 Materials Science and Technology Division, Los Alamos National Laboratory, Los Alamos, NM 87545, USA; 3 Stiftung Institut für Werkstofftechnik, Badgasteiner Str. 3, 28359 Bremen, Germany

## Abstract

Ion irradiation is an alternative to heat treatment for transforming organic-inorganic thin films to a ceramic state. One major shortcoming in previous studies of ion-irradiated films is the assumption that constituent phases in ion-irradiated and heat-treated films are identical and that the ion irradiation effect is limited to changes in composition. In this study, we investigate the effects of ion irradiation on both the composition and structure of constituent phases and use the results to explain the measured elastic modulus of the films. The results indicated that the microstructure of the irradiated films consisted of carbon clusters within a silica matrix. It was found that carbon was present in a non-graphitic sp^2^-bonded configuration. It was also observed that ion irradiation caused a decrease in the Si-O-Si bond angle of silica, similar to the effects of applied pressure. A phase transformation from tetrahedrally bonded to octahedrally bonded silica was also observed. The results indicated the incorporation of carbon within the silica network. A combination of the decrease in Si-O-Si bond angle and an increase in the carbon incorporation within the silica network was found to be responsible for the increase in the elastic modulus of the films.

Silicon oxycarbide, or black glass, consists of a chemical structure where silicon is simultaneously bonded to carbon and oxygen^1^. Depending on processing history and chemical composition, silicon oxycarbide materials are composed of different phases including silicon, silica, silicon carbide, and free carbon clusters^2^. Silica is the major phase in silicon oxycarbide and unlike other phases is always present in silicon oxycarbide materials. In sol-gel derived films, the Si-O-Si network develops in the early stages of the process and during gelation. Free carbon clusters exist in heat-treated silicon oxycarbides if there is excess carbon, i.e., more carbon than the amount required for saturation of the silicon valences. In silicon oxycarbides derived from the ion irradiation of sol-gel thin films, carbon clusters are the result of C-H bond breaking during ion irradiation, where free carbon atoms either bond with other carbon atoms to form clusters of free carbon or bond with silicon atoms to enter the silica network. Films with a high concentration of silicon and carbon and a low concentration of oxygen may exhibit silicon carbide. Similarly, the presence of a high concentration of silicon may lead to the formation of a separate silicon phase.

Silicon oxycarbide is traditionally made from controlled pyrolysis of hybrid organic-inorganic compounds containing elements including silicon, hydrogen, carbon, oxygen, and nitrogen^3^. However, it is possible to convert the films with ion irradiation instead of heat treatment^4^,^5^,^6^. Ion irradiation offers the advantage of controlled release of specific elements^7^,^8^ and previous work has shown increased hardness of ion-irradiated films when compared with heat-treated films^9^.

The microstructure of silicon oxycarbide ceramics produced by heat treatment has been extensively studied^2^,^10^,^11^,^12^, whereas investigation of ion-irradiated films has been limited. In addition, understanding of the effects of ion irradiation on the atomic structure and properties of the constituent phases of ion-irradiated silicon oxycarbides does not exist. An underlying assumption in previous studies on ion-irradiated silicon oxycarbide films^8^,^13^,^14^ has been that the atomic structure of the phases (e.g., Si-O-Si bond angle, configuration of carbon atoms in free carbon clusters) is identical in heat-treated and ion-irradiated films. It is further assumed that the effect of ion irradiation is limited to changes in the chemical composition and concentration of the phases. However, there are indications that the energy deposited during ion irradiation alters the atomic structure of individual phases including amorphous silica, carbon, and the SiO_x_C_y_ tetrahedra at the interface of carbon and amorphous silica. The present study focuses on an investigation of the changes in both the chemistry and atomic structure of the constituent phases, and the effects of microstructure, the phases present and their atomic structure, on the elastic moduli of ion-irradiated silicon oxycarbide thin films.

## Results and Discussion

### Chemical composition of the ion-irradiated films

**Figure 1:**
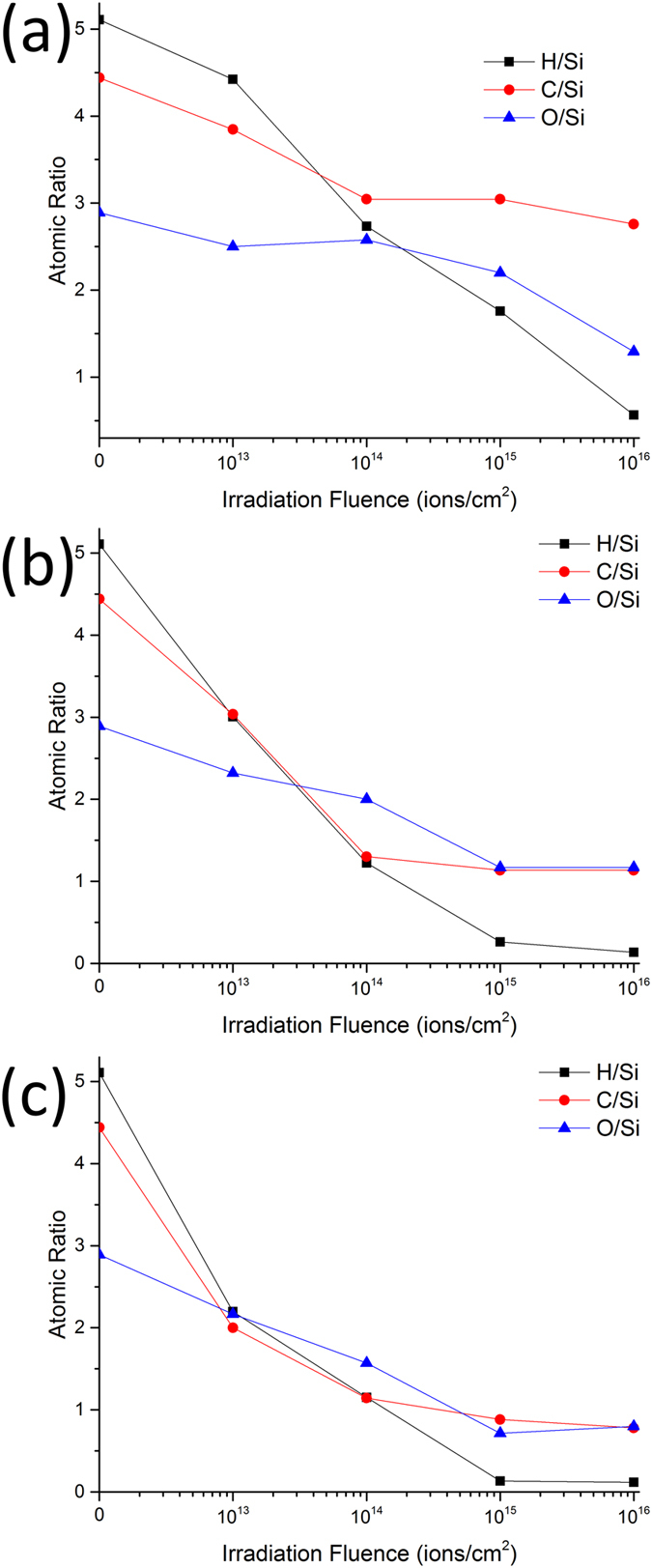
Variations in the atomic concentration ratio of carbon, oxygen, and hydrogen to silicon after irradiation with (**a**) 1 MeV Cu^+^, (**b**) 4 MeV Cu^2+^, and (**c**) 9 MeV Cu^3+^ ions.

Figure 1 shows the changes in the atomic concentration ratio of oxygen, hydrogen, and carbon when compared to silicon as a function of irradiation fluence for different ion energies, obtained from elastic recoil detection (ERD) and Rutherford backscattering spectrometry (RBS). A decrease in hydrogen, carbon, and oxygen concentration ratio is observed with increasing fluence for all ion energies. Hydrogen release is more pronounced than oxygen or carbon release, irrespective of the ion energy used. In general, ion irradiation of polymers typically leads to hydrogen loss^15^,^16^,^17^, as molecular hydrogen can escape the irradiation target easily due to the small size of the hydrogen molecules^18^. Some carbon loss is also expected due to the escape of carbon and hydrogen in the form of CH_4_^19^. However, oxygen loss and excessive carbon loss, while not unprecedented^20^,^21^,^22^,^23^, are not common. Previous studies on the bond dissociation energies of TEOS and similar compounds reveal that the dissociation of organometallic compounds to form oxygen containing gases requires higher energy than simply breaking C-H bonds^24^,^25^,^26^. This may explain the higher oxygen and carbon loss as a result of increasing ion energy and fluence, and thus an increase in the total deposited energy.

### Atomic structure of the free carbon clusters

**Figure 2:**
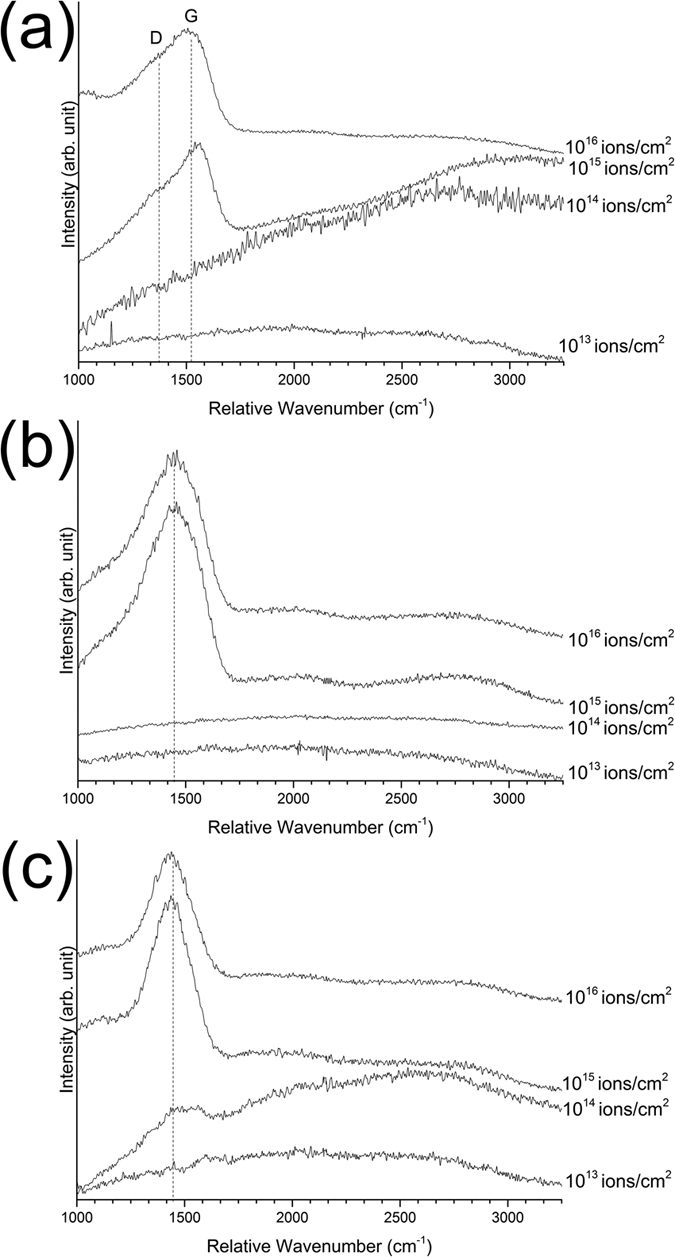
Raman spectra of ion-irradiated films after irradiation with (**a**) 1 MeV Cu^+^ (**b**) 4 MeV Cu^2+^ and (**c**) 9 MeV Cu^3+^ ions at different fluences.

Raman spectroscopy was performed to study the nature of the free carbon formed after ion irradiation. The Raman spectra of the green films were overwhelmed by a broad background emission, most probably due to intermediate organic materials. Figure 2(a) shows the Raman spectra of the films after irradiation with 1 MeV Cu^+^ ions. No Raman modes from the films are observed after irradiation with fluences of 10^13^ and 10^14^ ions/cm^2^. The background emission observed after irradiation with 1 MeV Cu^+^ ions with a fluence of 10^14^ ions/cm^2^ may have originated from intermediate organic compounds. The Raman spectra of the films after irradiation with 1 MeV Cu^+^ ions with fluences of 10^15^ and 10^16^ ions/cm^2^ exhibit carbon-related D and G modes, two broad modes centered at approximately 1350 and 1560 cm^−1^, as expected from the Raman spectra of amorphous carbon^27^,^28^. The strong background emission in the film irradiated with a fluence of 10^15^ ions/cm^2^ is related to the hydrogenation of free carbon clusters. The background signal originates from an electron hole pair recombination within sp^2^-bonded clusters, and the intensity of background emission is proportional to hydrogen concentration, primarily due to the saturation of non-radiative recombination sites (i.e., dangling bonds) by hydrogen^29^. The background emission disappears after irradiation with 1 MeV Cu^+^ ions with a fluence of 10^16^ ions/cm^2^, which is consistent with the reduced hydrogen concentration of the film after irradiation at this fluence (Fig. 1(a)). The Raman spectra of the films irradiated with 1 MeV Cu^+^ ions were fitted using two Gaussian peaks for the D and G modes. Following the method proposed by Ferrari *et al*.^27^, and based on the intensity ratio of the D mode to G mode and the spectral center of the G mode, the results indicate that the carbon clusters formed after irradiation are fully amorphous sp^2^-bonded carbon, where only a limited sp^3^-bonded carbon concentration might be present. Based on the lower G mode spectral center and D to G mode intensity ratio of the film irradiated with a fluence of 10^16^ ions/cm^2^, it can be stated that this film has a higher degree of damage in its graphitic six-fold rings than the film irradiated with a fluence of 10^15^ ions/cm^2^. However, no sign of sp^3^-bonded carbon is observed in either films.

Figure 2(b,c) show the Raman spectra of the films after irradiation with 4 MeV Cu^2+^ and 9 MeV Cu^3+^ ions. The films irradiated with a fluence of 10^13^ ions/cm^2^ exhibit no carbon-related Raman modes. No visible Raman mode is observed after irradiation with 4 MeV Cu^2+^ with a fluence of 10^14^ ions/cm^2^. After irradiation with 9 MeV Cu^3+^ ions with a fluence of 10^14^ ions/cm^2^ some evidence of carbon-related Raman modes is observed between 1100 and 1700 cm^−1^. However, the Raman modes are too weak to draw a conclusion about the nature of the free carbon clusters. The specimens irradiated with 4 MeV Cu^2+^ and 9 MeV Cu^3+^ ions with 10^15^ and 10^16^ ions/cm^2^ fluences exhibit a broad Raman mode centered at approximately 1430 cm^−1^ that cannot be deconvolved into D and G modes. Even though the amorphous carbon Raman response usually consists of D and G modes, there are cases where the Raman response of amorphous carbon is a single mode centered around 1430 cm^−1^ 30,31,32,33,34,35,36,37. This peak is usually observed in silicon carbide^30^,^31^,^33^,^35^ or germanium-carbon compounds^32^,^34^,^36^,^37^, and always in irradiated or sputtered specimens. The Raman mode was first observed by Gorman *et al*.^33^, in amorphous silicon carbide, and it was confirmed that this Raman mode originated from homonuclear carbon bonds. It has been attributed to both sp^2^-bonded carbon (but not in graphitic six-fold rings)^37^ and sp^3^-bonded^34^,^36^ carbon. Previous theoretical studies using an embedded-ring approach (ERA)^38^, and first principles density functional theory (DFT)^39^, have predicted a Raman mode at 1444 cm^−1^ due to either the 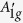 vibrational mode of a five or seven member sp^2^-bonded ring or linearly aggregated graphitic six-fold rings. However, since no additional D and G modes are observed, this mode has not originated from linearly aggregated graphitic rings. It should also be noted that no sp^3^-bonded carbon Raman mode is expected at wavenumbers higher than 1400 cm^−1^ 40. Thus, it can be concluded that the free carbon clusters formed in the films after irradiation with 4 MeV Cu^2+^ and 9 MeV Cu^3+^ ions are sp^2^-bonded carbon, arranged in a configuration other than graphitic six-member rings (e.g., five or seven member rings).

**Figure 3:**
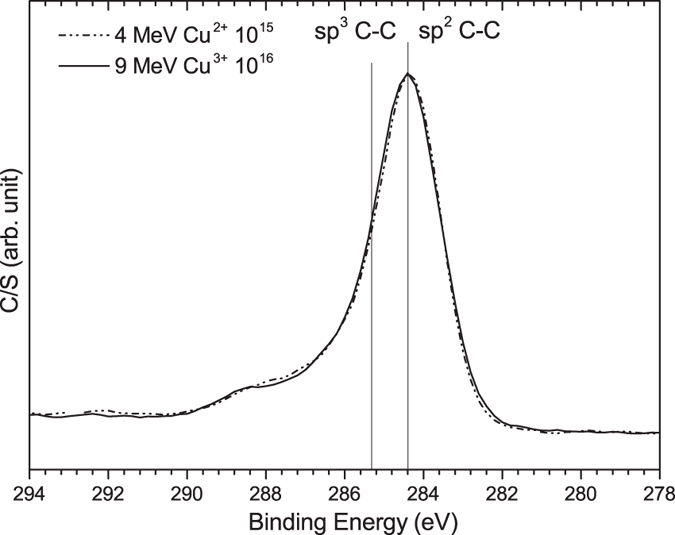
C 1s XPS spectra of the ion-irradiated films. The vertical lines indicate the position of sp2- and sp3-bonded carbon peaks.

The C 1s XPS spectra of the films can also be utilized to investigate the nature of bonding in free carbon clusters. Figure 3 shows the C 1s XPS spectra of the films after irradiation with 4 MeV Cu^2+^ ions with a fluence of 10^15^ ions/cm^2^ and 9 MeV Cu^3+^ ions with a fluence of 10^16^ ions/cm^2^. The C 1s spectra of both films consists of a main peak located at 284.4 eV, originating from C-C bonds, along with a shoulder at 287–290 eV due to C=O bonds^41^. The asymmetric broadening of the C-C peak is probably due to the contribution from the C-O-C peak at 286.5 eV^42^. The C 1s peaks of diamond (sp^3^-bonded) and graphite (sp^2^-bonded) are located at 285.3 eV and 284.4 eV, respectively^43^,^44^. By comparing the spectral center of the C-C peak with the location of the sp^2^- and sp^3^-bonded carbon, it is possible to identify the nature of free carbon and the concentration of sp^2^- and sp^3^-bonded carbon^43^,^44^. The results presented in Fig. 3 show that the carbon formed after irradiation with 4 MeV Cu^2+^ and 9 MeV Cu^3+^ ions is almost entirely sp^2^-bonded, further confirming the results from Raman spectroscopy that no sp^3^-bonded carbon has formed in the ion-irradiated films. The C-C peak in the C 1s spectra is not sensitive to the atomic configuration of carbon (i.e., five, six, or seven member rings) and so, the position of the peak is independent from the atomic configuration of sp^2^-bonded carbon.

### Evolution of the amorphous silica network

**Figure 4:**
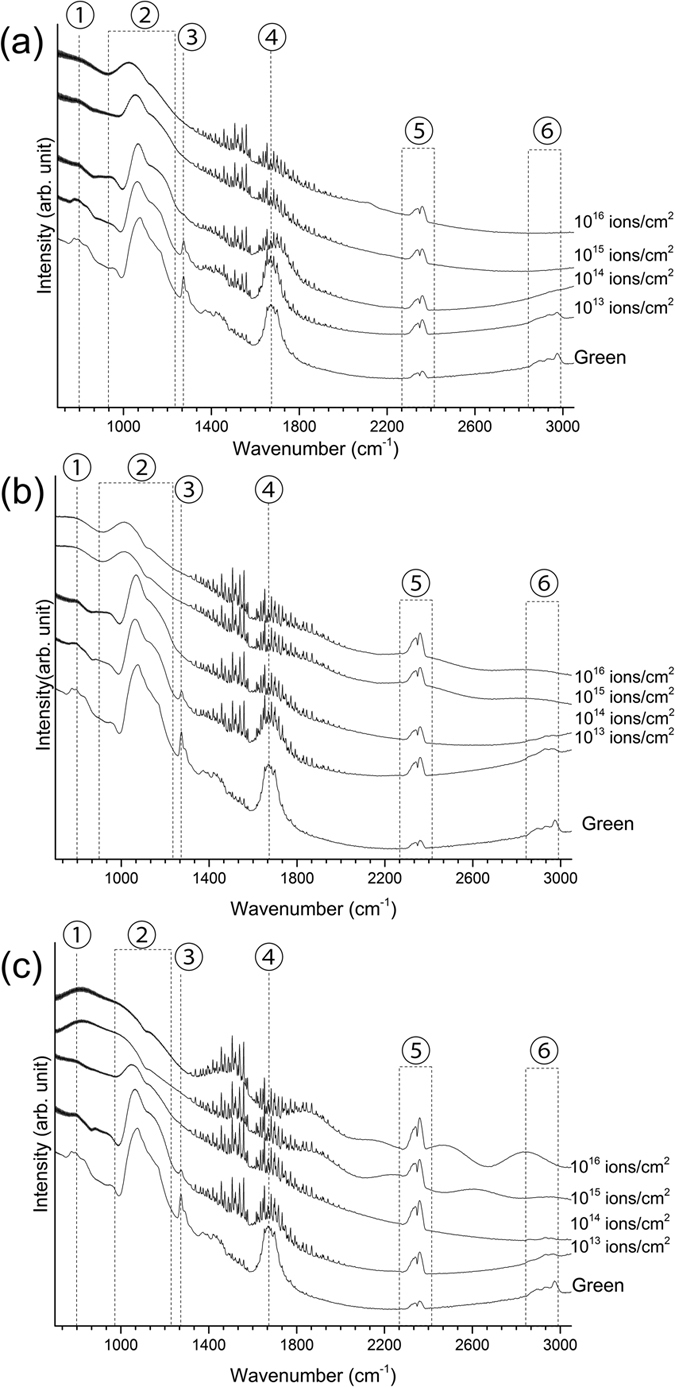
FT-IR spectra of films irradiated with different fluences with (**a**) 1 MeV Cu+, (**b**) 4 MeV Cu2+, and (**c**) 9 MeV Cu3+ ions. The peaks indicated on the figure are related to ①SiC-CH_3_ bonds, ②tetrahedrally bonded silica peaks (TO, LO, and O-Si-C peaks), ③C-H bonds in Si-CH_3_, ④O-H bonds, ⑤C-O bonds from carbon dioxide in the air, and ⑥C-H_x_ ( = 1,2,3) bonds.

Figure 4 shows the FT-IR spectra of the ion-irradiated films. The FT-IR spectra of the green film and films irradiated with lower fluences (10^13^ and 10^14^ ions/cm^2^) exhibit peaks related to organic materials. The FT-IR peaks at 750–800 cm^−1^, 1275 cm^−1^, 1450 cm^−1^, 1670 cm^−1^, and 2850 to 3000 cm^−1^ are related to the starting organic materials. The intensity of the peaks related to the organic materials decreases with increasing fluence for all ion energies. The decrease in the intensity of the mentioned peaks is consistent with the decrease in hydrogen concentration of the irradiated films (Fig. 1). The peaks between 2300 and 2500 cm^−1^ are related to C-O bonds from air and insufficient background subtraction. The fringe patterns between 1200–2000 cm^−1^ are related to atmospheric water vapor^45^.

There are three observable FT-IR peaks, related to the tetrahedral silica network, located between 900 and 1300 cm^−1^. In addition to the two main transverse optical (TO) and longitudinal optical (LO) peaks (located at approximately 1080 and 1200 cm^−1^, respectively), the introduction of carbon within the silica network and the formation of carbon-rich SiO_x_C_y_ tetrahedra (O-Si-C bonds) gives rise to an additional peak around 1140 cm^−1^ 46,47, manifested as the broadening and merging of the TO and LO peaks. The intensity ratio of the TO to LO peaks may be used as a measure of porosity^48^,^49^,^50^, where the LO peak is not expected in the absence of porosity. In addition, the spectral center of the TO peak is a measure of the Si-O-Si bond angle in silica, which by itself, is an indication of the structural compaction of the silica network (a decrease in free volume which results in an increase in density of the silica).

After irradiation with 1 MeV Cu^+^ ions with fluences of 10^13^ and 10^14^ ions/cm^2^, no significant changes in the silica-related FT-IR peaks of the films are observed. However, a further increase in the fluence leads to the development of an O-Si-C related peak and the elimination of the LO peak. After irradiation with the highest fluence (10^16^ ions/cm^2^), the position of the TO peak is shifted to 1030 cm^−1^. The Si-O-Si bond angle can be estimated from the TO peak position^51^,^52^ where the TO spectral center of 1030 cm^−1^ corresponds to a bond angle of 130°.

The decrease in the Si-O-Si bond angle after ion irradiation is related to the combined effects of thermal spikes, defined as the local melting and fast quenching of formed localized melt during ion irradiation, and induced defects (vacancies and interstitial atoms) during ion irradiation^53^,^54^,^55^,^56^. Thermal spikes cause local melting and a subsequent quenching that yields a more compact atomic structure (i.e., smaller bond angle)^55^. In addition, induced point defects during ion irradiation and a subsequent structural relaxation (re-orientation and re-bonding of tetrahedra^53^,^54^,^56^) also leads to a decrease in the Si-O-Si bond angle^53^,^54^. It should be noted that both experimental results^55^ and molecular dynamics simulations^53^,^54^ indicate that the atomic structure of silica compacted by irradiation with photons, electrons, neutrons, and ions is similar to the atomic structure of silica compacted by applied pressure. In addition, it has been shown that the vibrational states of silica after compaction with ion irradiation is similar to the vibrational states of silica after compaction with applied pressure^53^. In addition to irradiation induced effects, the introduction of carbon into the silica network, and the distortion of electron clouds and electrostatic forces also causes a decrease in the Si-O-Si bond angle^57^. For comparison, the TO peak spectral center for the film heat-treated at 800 °C in air was measured to be at 1080 cm^−1^, corresponding to a bond angle of 144°, which is the reported bond angle of fused silica.

Irradiation with 4 MeV Cu^2+^ ions with fluences of 10^13^ and 10^14^ ions/cm^2^ does not change the silica-related FT-IR peaks of the irradiated films. However, after ion irradiation with fluences of 10^15^ and 10^16^ ions/cm^2^, a decrease in the peak position of the TO peak, and the elimination of the LO peak is observed. The TO peak spectral center in the FT-IR spectrum of the 4 MeV Cu^2+^ irradiated film with a fluence of 10^16^ ions/cm^2^ shifts to 1015 cm^−1^. This spectral center corresponds to a Si-O-Si bond angle of 124°, indicating a more compact silica network and the presence of mainly three- and four-fold tetrahedra rings^58^.

After irradiation with 9 MeV Cu^3+^ ions at 10^15^ and 10^16^ ions/cm^2^, all the previously observed FT-IR silica peaks disappear. The only visible peak is a broad FT-IR peak between 600 and 900 cm^−1^ and a wavy background between 1300 and 3000 cm^−1^. The background pattern originates from the small thickness of the films and internal reflections that give rise to a wavy sinusoidal background pattern^59^. The broad peak between 600 and 900 cm^−1^ originates from the partial transformation of silica from tetrahedral to octahedral coordination. Williams and Jeanloz^60^ studied the effects of pressure on the infrared spectra and atomic structure of amorphous silica. They noted that with increasing pressure, the intensity of tetrahedrally coordinated silica (SiO_4_ tetrahedra) peaks around 1100 cm^−1^ (LO and TO peaks) was reduced significantly, relative to the intensity of the new peaks between 600 and 900 cm^−1^. The bending and stretching of O-Si-O bonds in octahedrally coordinated silica (SiO_6_ octahedras) has vibrational peaks in the same region. Other studies have also noted that with increasing applied pressure, the SiO_4_ tetrahedra peaks disappear and evidence of additional SiO_6_-related vibrational peaks are visible in the infrared spectra^61^,^62^. This phase transition of silica has been observed by both X-ray absorption^63^ and Raman spectroscopy^64^ at higher pressures. The shift from tetrahedral to octahedral coordination is gradual^65^,^66^,^67^, and so at the initial increase in coordination number, the FT-IR spectra of the silica is defined by a broad featureless peak between 600 and 900 cm^−1^ and the absence of SiO_4_-related peaks between 900 and 1300 cm^−1^. It should be noted that the broad featureless peak originates from the small size of octahedrally coordinated clusters^62^. It is also a feature of the FT-IR spectra of ion-irradiated films that the small size of the formed compounds may render the well-defined peaks into broad featureless peaks^68^.

The driving force behind this transformation under applied pressure has been attributed to the structural changes in silica to accommodate the compaction. As the pressure increases, the ring size of the silica tetrahedra decreases from six to three or four, but with further increase in applied pressure and a constant decrease in bond angle, the coordination number changes to a more efficient packing coordinate, i.e., octahedral, to accommodate the high pressure^64^. Irradiation with energetic ions and the resulting bond scission, disorder, and thermal spikes simulates the pressure induced changes. Irradiation with 1 MeV Cu^+^ and 4 MeV Cu^2+^ ions decreases the bond angle, in the same way that higher applied pressure results in a smaller bond angle, and after irradiation with 9 MeV Cu^3+^ ions, there is a change in the coordination number of silica to accommodate the higher compaction of the atomic structure.

**Figure 5:**
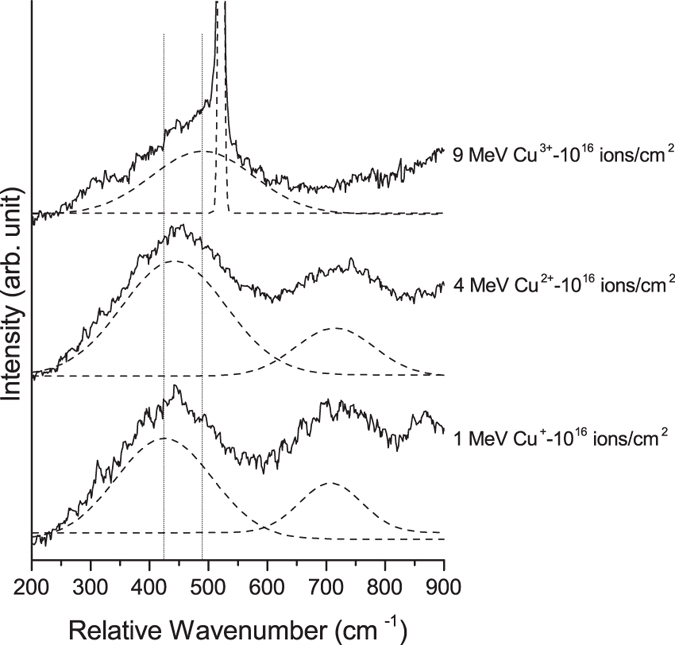
Raman spectra of the collected (solid line) and fitted (dashed line) silica Raman modes of the irradiated films. The vertical lines indicate the lowest and highest spectral center of the ω_1_ Raman mode. The sharp peak at 520 cm^−1^ originates from the silicon substrate. The substrate Raman mode was not observed for the films irradiated with 1 MeV Cu^+^ and 4 MeV Cu^2+^ which resulted in thicker films.

To further support this reasoning, the silica Raman modes were also investigated. Figure 5 shows the Raman spectra of the films after irradiation with a fluence of 10^16^ ions/cm^2^ for all ion energies investigated. The main two observable Raman modes are located at approximately 450 and 720 cm^−1^, corresponding to the ω_1_ Raman mode of amorphous silica, and carbon incorporated amorphous silica^69^, respectively. The Raman spectra were fitted with two Gaussian peaks. With increasing ion energy and irradiation fluence, there is an increase in the spectral center of the ω_1_ mode up to 490 cm^−1^ for the 9 MeV Cu^3+^ irradiated film with a fluence of 10^16^ ions/cm^2^. In addition, the 720 cm^−1^ mode disappears after irradiation at this fluence. The increase in the spectral center of the ω_1_ mode is similar to the results of previous studies on the Raman response of silica under pressure and is related to the decrease in the Si-O-Si bond angle and the formation of three- and four-fold tetrahedra rings^64^,^70^. The disappearance of the Raman mode at 720 cm^−1^ and a decrease in the intensity of the ω_1_ mode after irradiation with the 9 MeV Cu^3+^ ions is related to the changes in the coordination of silicon in amorphous silica. The presence of different coordinations and silicon sites results in broadening of the Raman modes and a decrease in intensity. In addition, the changes in coordination number may induce a decrease in the polarizability of the silicon and oxygen ions and a decrease in Raman intensity^60^. The Raman results confirm the FT-IR results which indicate that the silica network formed after ion irradiation is similar to the silica network after the application of high pressure.

**Figure 6:**
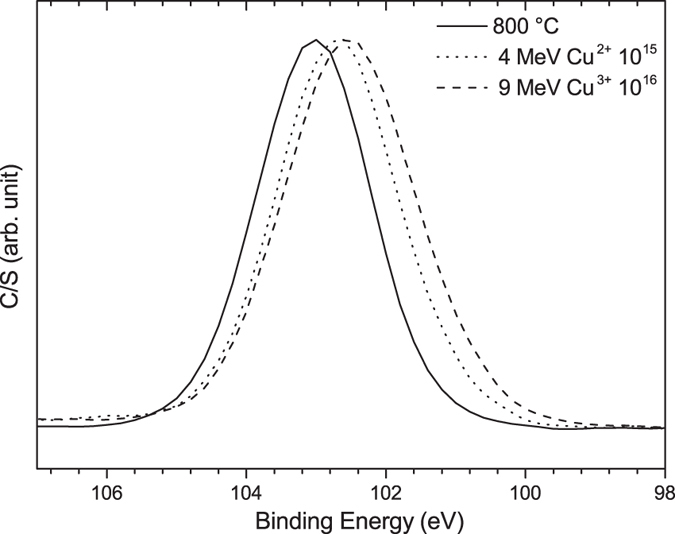
Si 2p core level XPS spectra of the ion-irradiated thin films.

In addition, XPS Si 2p core level spectra of the films after ion irradiation and heat treatment were also collected. Figure 6 shows the Si 2p core level spectra of the films after irradiation with 9 MeV Cu^3+^ ions with a fluence of 10^16^ ions/cm^2^ and 4 MeV Cu^2+^ ions with a fluence of 10^15^ ions/cm^2^. For comparison, the XPS spectrum of the heat-treated film at 800 °C is also included. The Si 2p spectra of silicon oxycarbide films after heat treatment can be considered as a combination of various SiO_x_C_y_ (SiO_4_, SiO_3_C, SiO_2_C_2_, SiOC_3_, as well as SiC_4_) tetrahedra^71^, with SiO_4_ positioned at 103.5 eV, SiC_4_ located at 100.3 eV, and the rest positioned in the middle. Any shift to lower binding energies indicates the incorporation of carbon in the silica network and the formation of carbon-rich SiO_x_C_y_ tetrahedra^71^,^72^,^73^. As shown in Fig. 6, after irradiation with 4 MeV Cu^2+^ ions with a fluence of 10^15^ ions/cm^2^, when compared to the heat-treated films, there is a shift toward lower binding energies and an asymmetric broadening of the peak. This is in agreement with the FT-IR results and indicates the incorporation of carbon within the silica network. A further shift toward lower binding energy is also observed after irradiation with 9 MeV Cu^3+^ ions with a fluence of 10^16^ ions/cm^2^. However, this shift cannot be solely related to carbon incorporation within the silica network, as the partial transformation of tetrahedrally to octahedrally coordinated silica also decreases the binding energy of silicon due to the higher extra-atomic relaxation energy in octahedrally coordinated silica in comparison with tetrahedrally coordinated silica^74^,^75^. It should be noted that based on the binding energy of the Si 2p peak, it can be concluded that the concentration of SiC in the films is negligible.

Both Raman and XPS results confirm the presence of a silica network within the films, even though silica-related FT-IR peaks between 900 and 1300 cm^−1^ are not observed after ion irradiation at the highest energy and fluences. In addition, the results also confirm a gradual transition toward a more compact silica network with increasing ion energy and fluence.

### Microstructure of the silicon oxycarbide thin films

There has been extensive discussion in the literature about the microstructural configuration of silica and carbon within silicon oxycarbides. Previous studies on heat-treated silicon oxycarbide materials have suggested two models for the microstructural configuration of carbon and silica: either an interconnected network of graphene or turbostratic carbon with silica nanodomains^76^,^77^ or a porous silica network with isolated free carbon clusters filling the voids^3^,^78^. However, the formation of an interconnected network in ion-irradiated films is unlikely. Ion irradiation deposits energy locally and further ion scattering may or may not lead to a near-homogenous distribution of energy within the irradiation target. Previous studies have confirmed that the microstructure of ion-irradiated polymer-derived silicon oxycarbide films consists of isolated carbon clusters oriented along the direction of the ion tracks within an amorphous silica network^14^,^79^,^80^. In addition, the lack of the carbon-related D and G modes in the Raman spectra of the films also indicates that the formation of an interconnected graphitic network is unlikely.

**Figure 7:**
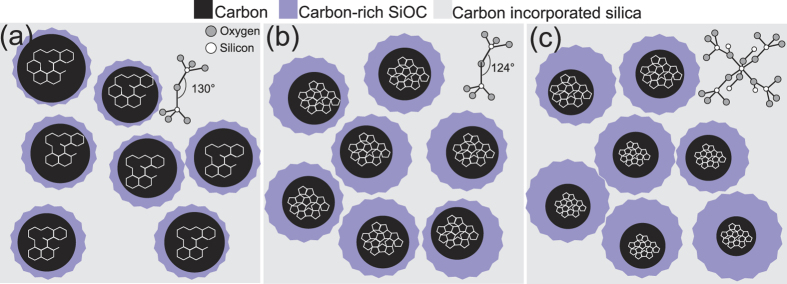
Microstructural configuration of films irradiated with different ion energies after irradiation with a fluence of 10^16^ ions/cm^2^, (**a**) 1 MeV Cu^+^, (**b**) 4 MeV Cu^2+^, and (**c**) 9 MeV Cu^3+^ ions.

A schematic of the proposed microstructure of the ion-irradiated films is shown in Fig. 7. The proposed microstructure of the film after ion irradiation at the highest fluence with 1 MeV Cu^+^ ions (Fig. 7(a)) consists of a compacted, tetrahedrally-bonded, and carbon incorporated amorphous silica network with average bond angle of 130°. In addition, isolated clusters of highly defective graphitic carbon are also present as a secondary phase within the films. The interface of silica and carbon is composed of carbon-rich SiO_x_C_y_ tetrahedra. The proposed microstructure of the film irradiated with 4 MeV Cu^2+^ ions at the highest fluence is presented in Fig. 7(b). The results indicate that the film is composed of a compact amorphous silica network along with isolated carbon clusters. The average Si-O-Si bond angle of the silica phase is estimated to be 124°. The carbon clusters are sp^2^-bonded carbon, arranged in a configuration other than graphitic six-fold rings, possibly five or seven member rings. The proposed microstructure of the film irradiated with 9 MeV Cu^3+^ ions at the highest fluence is presented in Fig. 7(c). The microstructure is composed of amorphous silica and isolated carbon clusters. The amorphous silica network is a combination of tetrahedrally and octahedrally bonded silica. The carbon atomic structure after irradiation with 9 MeV Cu^3+^ ions is sp^2^-bonded carbon, possibly arranged in five or seven member rings. The concentration of carbon-rich SiO_x_C_y_ tetrahedra in this film is also higher than other films.

### Elastic modulus of the ion-irradiated films

The force versus penetration depth curves obtained by the nanoindentation experiments were used to obtain the reduced elastic modulus of the films^81^. The initial portion of the unloading curve was fitted to a power law that allowed for the determination of the slope of the unloading curve at maximum depth (*h*_max_), i.e., stiffness (*S*). The value of *S* was then used to calculate *h*_*c*_, the contact depth of the indenter with the specimen using:


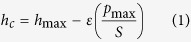


where *p*_max_ is the maximum load, and *ε* is a geometrical correction factor which depends on the geometry of the indenter and is 0.75 for a Berkovich indenter. The reduced elastic modulus of the film (*E*_*r*_) was then determined through knowledge of the projected area of the indenter (*A*), *h*_*c*_, and *S* using:


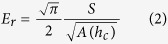


The elastic modulus (Young’s modulus) of the film is related to the measured *E*_*r*_ through:


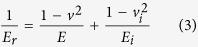


where *E* and *v* are the elastic modulus (Young’s modulus) and Poisson’s ratio of the film and *E*_*i*_ and *v*_*i*_ are the elastic modulus (Young’s modulus) and Poisson’s ratio of the indenter, respectively. With an estimate of the elastic modulus and Poisson’s ratio of the indenter and the Poisson’s ratio of the film, the elastic modulus of the film can be calculated. The elastic modulus and Poisson’s ratio of the diamond indenter are reported as 1140 GPa and 0.07, respectively^82^,^83^. Based on the reported Poisson’s ratio of graphene (0.165)^84^, SiC (0.19–0.29)^85^, and amorphous silica with different Si-O-Si bond angles (0.24–0.32)^66^, the Poisson’s ratio of the films was estimated to be 0.25. An estimated Poisson’s ratio between 0.2 and 0.3 affects the estimated elastic modulus by less than 3%.

**Figure 8:**
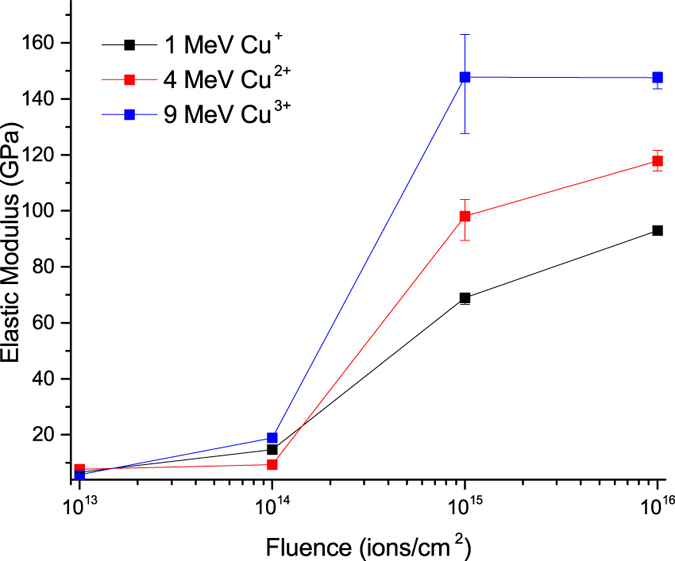
Elastic modulus of films after irradiation with 1 MeV Cu^+^, 4 MeV Cu^2+^, and 9 MeV Cu^3+^ ions at different fluences. Error bars represent the maximum and minimum values obtained.

Figure 8 shows the resulting elastic modulus of the films as a function of fluence for different ion energies. It is seen that after irradiation with a fluence of 10^16^ ions/cm^2^ for all ion energies used, the elastic moduli of the films are greater than that of fused silica. For example, the elastic modulus of the 9 MeV Cu^3+^ irradiated films with a fluence of 10^16^ ions/cm^2^ is double that of fused silica (147 GPa versus 72 GPa). The elastic moduli of the films are also larger than those usually reported in the literature for heat-treated SiOC ceramics in the absence of SiC (57–113 GPa^2^,^86^). Previous studies on the mechanical response of ion-irradiated silicon oxycarbide thin films have shown similar trends to those reported here, and the increase has been related to the formation of either diamond-like carbon or SiC^8^,^87^,^88^.

The secondary phase within the films in the present study has been determined to be sp^2^-bonded carbon, which reportedly does not affect the elastic modulus of silicon oxycarbides^89^. In addition, no sign of SiC was observed in the XPS, FT-IR, and Raman spectra of the films. Therefore other structural changes occurring after ion irradiation should be considered for the observed increase in the elastic modulus of the films. Additionally, the photoluminescence response of the 9 MeV Cu^3+^ irradiated film with a fluence of 10^16^ ions/cm^2^ was also investigated from room temperature down to 10 K, however no emission related to SiC (as observed previously in the irradiated silicon oxycarbide films^90^) was observed.

The decrease in the Si-O-Si bond angle of the silica after ion irradiation, in combination with the elastic deformation mechanism of amorphous silica, as discussed in more detail below, can explain the increase in the elastic modulus after ion irradiation. In addition to elastic deformation by pure bond length change, the presence of free volume in the amorphous silica network leads to an alternative elastic deformation mechanism^91^,^92^. This mechanism involves elastic deformation that is achieved not by pure bond length change, but through a small decrease in the bond angle of the bridging oxygen atoms and an increase in the packing of the amorphous silica. This alternative method can elastically deform the material at lower forces than by pure bond length change. As Si-O-Si bond angle decreases because of ion irradiation, this alternative mechanism becomes less prevalent and elastic deformation is mostly achieved through pure bond length change. This results in a higher elastic modulus of ion irradiated films. In general, any parameter that affects the bond angle of Si-O-Si including temperature and applied pressure^93^ affects the elastic modulus of amorphous silica. The level of enhancement depends on the bond angle and compaction of the atomic structure. An estimate of the elastic modulus of the silica phase can be made using the results of previous studies on the relationship between bond angle and applied pressure with elastic modulus^66^. Based on the Si-O-Si bond angle, the silica network in the films after irradiation with 1 MeV Cu^+^ and 4 MeV Cu^2+^ ions with a fluence of 10^16^ ions/cm^2^ is similar to a silica network under applied pressures of 9 and 17 GPa, respectively. For applied pressures of 9 and 17 GPa, elastic moduli of 94 GPa and 107 GPa were predicted, respectively. In the present study, the measured elastic moduli of the films after irradiation with 1 MeV Cu^+^ and 4 MeV Cu^2+^ ions with a fluence of 10^16^ ions/cm^2^ were 93 and 118 GPa, respectively. After irradiation with 9 MeV Cu^3+^ ions with a fluence of 10^16^ ions/cm^2^ it has been shown that the coordination number of silica begins to change. This change in coordination number for silica under pressure occurs at 20–25 GPa^66^,^67^. This pressure range corresponds to a predicted elastic modulus of 129 GPa. This estimate of the elastic modulus of the silica phase is comparable to the measured elastic modulus of 147 GPa for the film irradiated with 9 MeV Cu^3+^ with a fluence of 10^16^ ions/cm^2^. It should be noted the increase in the elastic modulus of the 9 MeV Cu^3+^ irradiated film with a fluence of 10^16^ ions/cm^2^ is related to both the decrease in Si-O-Si bond angle and the formation of octahedrally bonded silica. The elastic modulus of the stishovite, the high pressure octahedrally coordinated phase of silica is reported to be 400 GPa^94^, and thus the formation of octahedrally coordinated clusters of silica is expected to enhance the elastic modulus of the films.

In addition, the increase in the concentration of carbon-rich SiO_x_C_y_, as inferred from the XPS and FT-IR results, also aids in increasing the elastic modulus of the films. Considering the agreement between the estimated modulus of the silica phase and the measured moduli of the films, the effect of the increase in the concentration of carbon-rich SiO_x_C_y_ most likely plays a minor role.

The higher measured elastic modulus of the 1 MeV Cu^+^ irradiated films when compared with fused silica is related to an ion irradiation induced decrease in the Si-O-Si bond angle. The higher elastic moduli of the films after irradiation with 4 MeV Cu^2+^ ions is related to a further decrease in the Si-O-Si bond angle. Finally, the increase in the elastic moduli of the films irradiated with 9 MeV Cu^3+^ ions is attributed to both a decrease in the Si-O-Si bond angle and the formation of pockets of octahedrally coordinated silica. The formation of carbon-rich SiO_x_C_y_ tetrahedra may also contribute to the increase in elastic modulus, but its effect is secondary to that caused by changes in the Si-O-Si bond angle.

## Conclusions

The chemical and microstructural evolution of the films after ion irradiation resulted in a microstructure composed of an amorphous silica network and free carbon clusters. The chemical composition of the irradiated films, derived from ERD/RBS, indicated complete hydrogen depletion, as well as extensive carbon and oxygen loss after ion irradiation. Based on Raman spectroscopy and XPS results, the free carbon clusters were found to be sp^2^-bonded, although after irradiation at higher energies the carbon clusters were arranged in non-graphitic configurations. In addition, FT-IR and Raman spectroscopy results indicated that there was a monotonic reduction in the Si-O-Si bond angle with increasing fluence or ion energy, and after irradiation with 9 MeV Cu^3+^ ions, pockets of octahedrally coordinated silica were formed. An increase in the concentration of carbon rich SiO_x_C_y_ tetrahedra with increasing fluence or ion energy was also observed in both XPS and FT-IR spectroscopy results. Changes in the Si-O-Si bond angle and the formation of carbon rich SiO_x_C_y_ tetrahedra were found to be responsible for the observed increase in the elastic moduli obtained by nanoindentation.

## Methods

### Synthesis of sol-gel films

The films were synthesized through a sol-gel method, as previously described^5^. A mixture of 7.5 mol ethanol, 1 mol distilled water, and 1 mol acetic acid was stirred at room temperature in a beaker, followed by sequential addition of 0.6 mol methyltriethoxysilane (MTES) and 0.4 mol tetraethylorthosilicate (TEOS), drop by drop under vigorous stirring. The solution was stirred for 10 min before adding 0.25 mol polyvinylpyrrolidone (PVP). The mixture temperature was then raised to 50 °C and kept at this temperature for 30 min. Prior to deposition, the sol was stored for 24 h in an argon atmosphere and was then spin-coated onto a polished (100) silicon wafer. The films were dried at 80 °C and heat-treated at 300 °C in air for 30 mins. The film thickness was measured by a step height technique using an atomic force microscope. The thickness of the films after deposition was close to 1000 nm, and ranged from 200 nm to 1000 nm after ion irradiation.

### Ion irradiation

Ion irradiation was performed using a 3 MV tandem accelerator to produce 1 MeV Cu^+^, 4 MeV Cu^2+^, and 9 MeV Cu^3+^ ions with fluences from 1 × 10^13^ to 10^16^ ions/cm^2^. The projected ion range for all irradiations was larger than the film thickness. In order to avoid thermal effects during irradiation, the beam current was kept relatively low, ~0.5 μA/cm^2^. For comparison, one film was also heat-treated at 800 °C in air for 30 min.

### Rutherford backscattering spectrometry and elastic recoil detection

Rutherford backscattering spectrometry (RBS) was performed using a 3.83 MeV ^4^He^+^ ion beam. The backscattered ions were collected using a silicon surface barrier detector at the Cornell geometry with a scattering angle of 167°. Elastic recoil detection (ERD) experiments were performed using a 2 MeV ^4^He^+^ ion beam with an incident angle of 75° in the IBM geometry, and the recoiled hydrogen atoms were collected at a scattering angle of 30°.

### Raman, FT-IR, and X-ray photoelectron spectroscopy

Raman spectroscopy was performed using a WITec confocal microscope, and a 532 nm Nd:YAG laser as the light source. The light was focused onto the surface of the films using a 100X/0.9NA objective, and the scattered light was collected using the same objective. The collected light was then focused onto a 100 μm diameter optical fiber which acted as the confocal pinhole. The light was dispersed using either an 1800 g/mm or 600 g/mm grating and was detected by a CCD camera. Each reported Raman spectrum is the average of five separate spectra collected on different locations. Fourier transform infrared (FT-IR) spectroscopy was performed in transmission using an Agilent 680 IR spectrometer in the range of 400 to 4000 cm^−1^ with 4 cm^−1^ resolution. The reported data is the average of sixteen separate collections. X-ray photoelectron spectroscopy (XPS) was performed using a PHI Quantera SXM scanning XPS microprobe. Al-Kα radiation (1486.6 eV) was used as the X-ray source. No ion etching was performed prior to the measurements and charge correction was performed automatically by controlled electron irradiation.

### Nanoindentation

The reduced elastic modulus of the films was obtained by performing nanoindentation experiments using a load-controlled commercial nanoindenter with a diamond Berkovich indenter tip. The instrument compliance and indenter area function were obtained by performing indentations in fused silica and tungsten using the procedure of Oliver and Pharr^82^. Prior to performing the experiments, the instrument and specimen were allowed to thermally equilibrate for 10–12 h in a thermal enclosure. The experiments were performed with a linear loading function of 10 s loading, 20 s holding at the maximum load, and 10 s unloading. The reported data for the indentations represent the average of five experiments.

## Additional Information

**How to cite this article**: Shojaee, S.A. *et al*. Ion irradiation induced structural modifications and increase in elastic modulus of silica based thin films. *Sci. Rep.* **7**, 40100; doi: 10.1038/srep40100 (2017).

**Publisher's note:** Springer Nature remains neutral with regard to jurisdictional claims in published maps and institutional affiliations.
